# “We are human beings, we also get sick”: presenteeism in nursing workers in a pandemic context

**DOI:** 10.1590/1518-8345.6861.4053

**Published:** 2023-11-03

**Authors:** Tanyse Galon, Vera Lucia Navarro

**Affiliations:** 1 Universidade Federal do Triângulo Mineiro, Instituto de Ciências da Saúde, Departamento de Enfermagem na Assistência Hospitalar, Uberaba, MG, Brasil.; 2 Universidade de São Paulo, Faculdade de Filosofia, Ciências e Letras de Ribeirão Preto, Departamento de Psicologia, Ribeirão Preto, SP, Brasil.

**Keywords:** Presenteeism, Occupational Health, Working Conditions, Nursing Team, Pandemics, COVID-19, Presentismo, Salud Laboral, Condiciones de Trabajo, Grupo de Enfermería, Pandemias, COVID-19, Presenteísmo, Saúde Ocupacional, Condições de Trabalho, Equipe de Enfermagem, Pandemias, COVID-19

## Abstract

**Objective:**

to understand the experiences of presenteeism in nursing professionals from hospital services during the COVID-19 pandemic.

**Method:**

qualitative study, anchored in historicaldialectical materialism. Thirty nursing workers participated in the research, divided into six online focus groups, analyzed based on Hermeneutics-Dialectics.

**Results:**

three categories of analysis emerged: “Worsening presenteeism in the pandemic context”; “Why did I go to work sick: the worker’s decision or precarious work?”; “Old problems, permanent struggle”. Despite the illness of professionals by COVID-19, presenteeism in the pandemic was marked by institutional pressure to return to work, mental suffering and lack of recognition and humanization. Among the factors that led to presenteeism, the lack of testing for COVID-19, concern for patients, co-workers and managers, as well as fear of losing their job and/or financial benefits, stood out. Faced with this scenario, workers called for a new reality in which rights such as decent wages and safe working conditions are guaranteed.

**Conclusion:**

the pandemic context revealed a worsening of presenteeism among nursing professionals. The results pointed to the importance of concretely valuing nursing in legal terms and beyond honors.

## Abstract (es)

**Objetivo:**

comprender las experiencias de presentismo vividas por profesionales de enfermería en los servicios hospitalarios durante la pandemia de COVID-19.

**Método:**

estudio cualitativo, anclado en el materialismo histórico-dialéctico. Participaron de la investigación treinta trabajadores de enfermería, divididos en seis grupos focales en línea, analizados con base en la Hermenéutica-Dialéctica.

**Resultados:**

emergieron tres categorías de análisis: “Agravamiento del presentismo en el contexto de pandemia”; “¿Por qué fui a trabajar enfermo: decisión del trabajador o trabajo precario?”; “Viejos problemas, lucha permanente”. A pesar del padecimiento de los profesionales por la COVID-19, el presentismo en la pandemia estuvo marcado por la presión institucional para regresar al trabajo, el sufrimiento mental y la falta de reconocimiento y humanización. Entre los factores que propiciaron el presentismo destacaron la falta de pruebas de COVID-19, la preocupación por los pacientes, compañeros de trabajo y gestores, así como el miedo a perder su empleo y/o beneficios económicos. Ante este escenario, los trabajadores reclamaron una nueva realidad en la que derechos como salarios dignos y condiciones laborales seguras estén garantizados.

**Conclusión:**

el contexto de pandemia reveló un agravamiento del presentismo entre los profesionales de enfermería. Los resultados apuntaron la importancia de valorar concretamente la enfermería en términos legales y más allá de los honores.

## Abstract (pt)

**Objetivo:**

compreender as experiências de presenteísmo vivenciadas por profissionais de enfermagem de serviços hospitalares durante a pandemia da COVID-19.

**Método:**

estudo qualitativo, ancorado no materialismo histórico-dialético. Participaram da pesquisa 30 trabalhadores da enfermagem, distribuídos em seis grupos focais online, analisados a partir da Hermenêutica-Dialética.

**Resultados:**

emergiram três categorias de análise: “Agravamento do presenteísmo no contexto pandêmico”; “Por que fui trabalhar doente: decisão do trabalhador ou precarização laboral?”; “Velhos problemas, luta permanente”. Apesar do adoecimento de profissionais pela COVID-19, o presenteísmo na pandemia foi marcado pela pressão institucional para retorno ao trabalho, sofrimento mental e falta de reconhecimento e humanização. Dentre os fatores que levaram ao presenteísmo, destacaram-se a falta de testagem para a COVID-19, a preocupação com pacientes, colegas de trabalho e gestores, bem como o medo de perder o emprego e/ou benefícios financeiros. Diante desse cenário, os trabalhadores clamaram por uma nova realidade na qual direitos como salários dignos e condições seguras de trabalho sejam garantidos.

**Conclusão:**

o contexto pandêmico revelou um agravamento do presenteísmo entre os profissionais de enfermagem. Os resultados apontaram para a importância da valorização concreta da enfermagem em termos legais e para além das homenagens.

**Highlights:**

**(1)**Institutional pressure, mental suffering and devaluation are marked by presenteeism.

**(2)** Lack of access to COVID-19 screening tests resulted in presenteeism.

**(3)** Concern for patients, coworkers, and managers resulted in presenteeism.

**(4)** Fear of losing employment or financial benefits resulted in presenteeism

**(5)** It is urgent to value nursing, beyond speeches and tributes.

## Introduction

Presenteeism is defined as the act of working while ill or being present in the workplace under conditions that would require the absence of the worker ^(^ 1 ^)^. Nursing professionals, whose activity involves providing care and concern for the well-being of individuals and populations, are subject to working even when they are sick ^(^ 1 ^-^ 2 ^)^. The global prevalence of presenteeism among nursing workers, estimated at 49.2%, is alarming and evokes the need for a careful look at this phenomenon ^(^ 2 ^)^. 

Health emergency scenarios can lead workers to an increase in presenteeism, due to factors such as a sense of responsibility, institutional pressure to be present and lack of substitutes in case of absence from work ^(^ 3 ^)^. During the 2019 coronavirus disease (COVID-19) pandemic, health professionals, including nursing workers, could not benefit from the “stay at home” recommendation and were exposed to intense contamination and death from the disease ^(^ 4 ^-^ 5 ^)^. 

In Brazil, data from the Federal Nursing Council (COFEN) showed that until April 17, 2023, approximately 65,000 cases of COVID-19 were reported among Brazilian nursing professionals, with approximately 872 deaths from the disease ^(^ 6 ^)^, reflections of a country that came to occupy the first position in the world in cases of death of these workers in the pandemic ^(^ 7 ^)^. 

It is important to highlight that nursing professionals already faced a historical and chronic precariousness of work that was not inaugurated, but aggravated by the pandemic context ^(^ 8 ^)^. Even so, researchers and health professionals have denounced the numerous government failures and negligence in dealing with the COVID-19 pandemic in Brazil, which negatively affected the health and safety of nursing professionals ^(^ 9 ^-^ 12 ^)^. 

The management of the pandemic crisis by the Brazilian Federal Government was marked by discourses that denied the disease, lack of articulation between the three levels of the Unified Health System ( *Sistema Único de Saúde-*SUS), high public spending on treatments without scientific proof, lack of systematic tracking and testing, weaknesses in case and death records, in addition to negligence and delay in purchasing vaccines ^(^ 9 ^-^ 12 ^)^. This panorama directly and indirectly influenced the increase in cases of contamination and death of the Brazilian population, the collapse of health services and, consequently, the overload and illness of nursing professionals, who continued to work despite daily exposure to the virus and intense suffering physical and mental ^(^ 9 ^-^ 12 ^)^. 

In addition, the act of working while sick increases the risk of failures in patient safety, a decrease in the quality of care and an increase in financial costs for health institutions, arguments that also support the importance of studying, understanding and debating this issue ^(^ 13 ^)^. 

Therefore, this study aimed to understand the experiences of presenteeism in nursing professionals from hospital services during the COVID-19 pandemic.

## Method

### Type of study

This is a descriptive, exploratory research with a qualitative approach. From the perspective of historical-dialectical materialism, the analysis considered the logic of the capitalist mode of production as a foundation in the production of various forms of precarious work processes, generating workloads and wear and tear on the physical and mental health of workers ^(^ 14 ^-^ 15 ^)^. 

The construction of the manuscript followed the Consolidated Criteria for Reporting Qualitative Research (COREQ), meeting the scientific requirements for a qualitative study.

### Research scenario

The research was carried out with nursing professionals from two Brazilian municipalities, one located in the countryside of the state of São Paulo (SP) and the other in the interior of the state of Minas Gerais (MG). Both are considered regional hubs in health care, which allowed access to research participants, namely nursing professionals working in hospital sectors during the COVID-19 pandemic.

### Period

Data collection was carried out between June 2020 and October 2021.

### Study participants and selection criteria

The study included 30 nursing professionals, 12 nurses and 18 nursing technicians from the public and/or private hospital network in two cities in the interior of the state of São Paulo and Minas Gerais, in professional practice for at least six months and who experienced one or more episodes of presenteeism during the COVID-19 pandemic.

The selection of participants was based on snowball strategy ^(^ 16 ^)^ where the researchers contacted, via email or communication applications, initial informants (nursing professionals), who in turn indicated other possible participants. This process gave rise to a network of contacts that was interrupted when the data obtained became redundant or repetitive, through sampling by theoretical saturation ^(^ 17 ^)^. 

The authors opted for a data collection that did not involve looking for hospitals to carry out the study, as it is a topic that could generate embarrassment, exposure, insecurity, fear or bias among workers when talking about presenteeism in these spaces. Thus, we worked with the snowball strategy, in direct contact with the participants, so that such situations were minimized.

### Data collection instruments

Online focus groups were conducted based on a semi-structured script prepared by the authors, containing seven guiding questions: “1. What does it mean to you to be a nursing professional in this context of the COVID-19 pandemic? 2. What work experience most impacted you during the pandemic? 3. Has your work during the pandemic affected your physical and/or mental health? if so, in what way? 4. How were your work relationships during the pandemic? 5. Have you ever gone to work sick during the pandemic? Tell me one or more experiences; 6. What factors led you to work even though you were sick or not feeling well? 7. What strategies should be developed so that nursing workers have better working and health conditions?”. This script was built based on the scientific literature and evaluated by three specialist researchers in the subject involved, resulting in the final version.

### Data collection

Six online focus groups ^(^ 18 ^-^ 19 ^)^ were organized with five nursing professionals in each group. The number of participants in each focus group was defined based on the recommendations of the scientific literature ^(^ 19 ^)^. The option to form smaller groups also provided a more suitable space for further discussions and acceptance of a sensitive topic, which brought out memories of a strong emotional nature. This reception process could be observed in the groups throughout the research, carried out not only by the researchers, but also among the workers themselves, in sharing their experiences. 

The online focus group strategy was selected considering that data collection took place in the context of the COVID -19 pandemic, which required social distancing in the face of contamination risks, in addition to work overload, variations in work shifts and complex work protocols to be fulfilled by nursing professionals, aspects that prevented face-to-face data collection.

The online focus groups, scheduled by telephone and conducted by one of the authors, were carried out in a private virtual environment using WhatsApp ^®^, a free communication application accessible to participants. The groups were formed in order of membership: the first five professionals who agreed to participate in the research made up the first group, and so on. The groups were developed asynchronously, that is, members were not required to have a simultaneous connection ^(^ 18 ^)^. This strategy is recommended when participants have different schedules available, a common situation among nursing professionals. 

The author who moderated the groups asked a question a day and mediated the discussions in search of the expression of presenteeism experiences and the exchange of reflections among the participants. At the end of seven days, the groups were closed and deactivated with their knowledge and authorization, with a view to preserving the secrecy and confidentiality of the data.

### Data treatment and analysis

Hermeneutics-Dialectics ^(^ 20 ^)^ was adopted, based on the following analytical steps: a) Ordering of data, with transcription of reports and primary reading of all material, enabling a horizontal panorama of findings; b) Classification of the data, with exhaustive and repeated reading of the texts, the so-called “floating reading”, aiming to apprehend the structures of relevance and the central ideas transmitted by nursing professionals. At the end of this step, a transverse reading of each online focus group was carried out, producing units of meaning, which in turn were allocated and grouped into themes or categories, in search of the construction of an analysis system; c) Final analysis or interpretative synthesis, with the objective of articulating analytical and empirical categories through a dialectical movement between the experiences of presenteeism reported by the workers and the theoretical framework of historical-dialectical materialism. 

### Ethical aspects

The research followed the recommendations of Resolution nº 466/2012 of the National Health Council (CNS) and was approved by the Research Ethics Committee of the *Universidade Federal do Triângulo Mineiro* with Certificate of Presentation for Ethical Appreciation (CAAE) nº 82365417.9.0000.5154, opinion No. 2,543,320. All study participants signed the Informed Consent Form (ICF). In the description of the results, aiming at preserving anonymity, the abbreviations G for group, P for participant, NUR for nurse and NT for nursing technician were used. 

## Results

Of the 30 participants, 12 were nurses and 18 were nursing technicians, 22 women and eight men, aged between 22 and 51 years. Of the total number of respondents, 18 had only one job and 12 reported more than one job in nursing, with 14 working only in private services, 13 in public services and three in both contexts.

**Figure 1 -:**
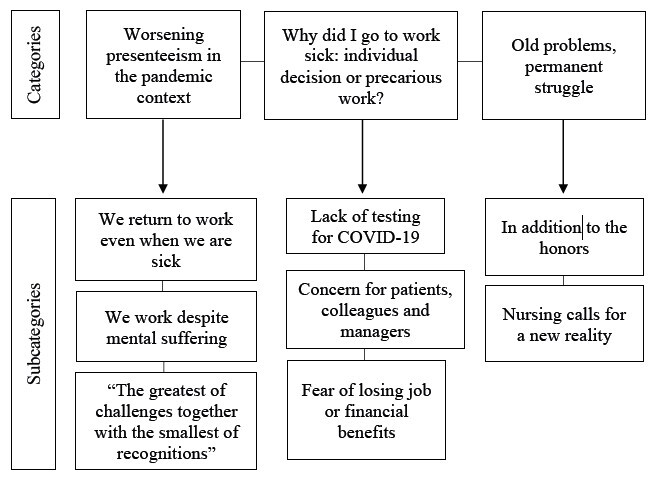
Categories and subcategories developed from the analysis of presenteeism in nursing professionals from hospital services during the COVID-19 pandemic (N=30). Brazil, 2020-2021

Based on the research question “What were the experiences of presenteeism lived by nursing professionals from hospital services during the COVID-19 pandemic?” three thematic categories and eight subcategories emerged, shown in Figure 1. 

### Worsening presenteeism in the pandemic context

#### We return to work even when we are sick

Most of the professionals interviewed reported suspected or confirmed diagnosis of COVID-19 during the pandemic. Living with teammates being hospitalized as a result of COVID-19 was recurrently reported, which also showed the severity with which the disease affected nursing professionals. Despite this, reports of institutional pressure were identified to return to work without the professionals being fully recovered. Workers with flu-like symptoms and a subsequent negative result for COVID-19 reported even greater pressures for presenteeism, although they were also unable to return to work.

*When I came back, it wasn’t completely healed... even so, they released me for work and didn’t do any more tests. (...) Yesterday it was four months since I got infected, I still can’t fully feel the taste, I have headache several times a week.* (G3, P3, NT) 

*I heard people who, even after isolation, felt fatigue, saying that they were still not 100%.* (G6, P5, NUR) 

*This week at the hospital a nursing technician went to work with symptoms of COVID-19. He stayed away for a few days, he tested positive. Today I heard him coughing. They reported that the employee’s doctor has already released him from isolation. (...) We have suspected or positive patients and two employees hospitalized with COVID-19, one of whom was intubated this afternoon.* (G6, P4, NUR) 

*During the pandemic, I had some symptoms and the doctor took me away for seven days. (...) The doctor from the SESMT [Specialized Service in Safety Engineering and Occupational Medicine] called me to return to work since my exam was negative for COVID-19 and that I had a flu syndrome (common cold). (...) This demonstrates the exploitation that nursing professionals are subjected to, even more so in times of a pandemic, as she did not even ask me if I was better, she just said that with the negative test I was able to work.* (G3, P2, NT) 

*Being a nursing professional in the pandemic meant giving up your rest, your family, and life itself, because “if you didn’t catch the virus, then you’re healthy to work”.* (G4, P3, NT) 

#### We work despite mental suffering

According to participants, presenteeism was linked to mental distress throughout the pandemic. Working under strong pressure and intense emotional overload were common situations in the face of the high and complex demand for care, the shortage of professionals, the uncertainties facing a new disease, the fear of contaminating family members, social isolation, in addition to constant contact with the illness. and the death of patients and co-workers.

*I personally find myself feeling some types of anxiety that I didn’t have before the pandemic. It’s giving me a lot of stress.* (G3, P5, NT) 

*(...) The fear of transmitting it to my family is enormous! I see exhausted professionals, many distancing and overloading the others... the psychologically extremely shaken, at the same time having to be strong to help patients.* (G4, P4, NT) 

*We have a colleague who is working with post-COVID-19 depression.* (G5, P1, NUR) 

*My mental health was greatly affected by fatigue, lack of time for myself, distance from family, lack of social life, pandemic and overwork. Being away from friends, being afraid of bringing the virus into the house and bringing death to family members, is really something that touches your emotions.* (G4, P3, NT) 

*(...) Not only me but my colleagues live day after day emotionally overloaded. Dark circles, fatigue, crying (from many).* (G4, P1, NT) 

*I think mental pain is worse than physical pain (...). For me to put patients in bags, without the right to dignified clothes or a decent burial, was very difficult.* (G6, P2, NT) 

*In the psychological part, the sadness at seeing so many patients dying. Finally, seeing so many friends who worked with me being intubated.* (G4, P5, NT) 

#### “The greatest of challenges together with the smallest of recognitions”

In all discussion groups, nursing professionals expressed the close relationship between presenteeism, devaluation of the profession and dehumanization, brutally exacerbated during the COVID-19 pandemic. The lack of recognition by the employing institutions and the population served was reported, which produced a collective feeling of dehumanization at work when they were seen as “robots” who could not get sick.

*It’s been six years with the company and they haven’t changed this concept, that an employee cannot get sick.* (G1, P1, NT) 

*The most striking thing is that you are not recognized for this, for seeing your effort even when you are sick going to work, so as not to burden anyone! Not that everything we do needs a medal or a slap on the back, but being recognized for what you do and the importance you are giving to your work!* (G2, P3, NT) 

*When I was admitted to the hospital, we had a light makeup class to always look good and healthy, because patients think that we don’t get sick, we have to always look good, presentable, like robots and not people. (...) But we are human beings, we also get sick.* (G1, P4, NT) 

*(...) I worked seven days walking like a robot. They think we’re a machine, we just work, we can’t feel anything...* (G2, P2, NT) 

*Having your rest or restoring your physical or mental health is seen negatively by managers and colleagues, because in that moment you are needed, the late effects now do not matter.* (G4, P3, NT) 

*Working in the pandemic was the biggest challenge along with the smallest recognition. In my experience, I, a recent mother with a six-month-old baby and having to assume an ICU [Intensive Care Center] COVID-19 was something extremely frightening. I saw a huge lack of humanization towards the employee himself.* (G4, P1, NT) 

### Why did I go to work sick: the worker’s decision or precarious work?

#### Lack of testing for COVID-19

Participants reported cases of professionals who came to work with signs and/or symptoms suggestive of COVID-19, but without access to a permanent and organized institutional testing system and, therefore, without guarantee of a definitive diagnosis, a situation that generated uncertainties about their own illness, in addition to the constant fear of contaminating teammates, patients and family members.

*I have friends who suspected COVID-19 and were sick for two shifts and then left because they really couldn’t afford it. The tests hadn’t arrived*. (G1, P1, NT) 

*I had contact with a doctor who had to be isolated on suspicion of COVID-19! After a week I had a scratchy throat, but no other symptoms! Due to the lack of testing and seasonal illnesses, we were in doubt as to whether we should really walk away.* (G1, P3, NUR) 

*We have an employee who presented symptoms and the doctor who attended her gave her a five-day certificate and asked her to assess whether the symptoms worsened after the fifth day, however, she “improved” and went back to work. Okay, it might not be COVID-19, but it might as well be. This doubt, which everyone has, makes the situation much worse. It would be great if they had more rapid tests, especially for health workers. Thinking that if she has it, she will pass it on to all of us and all patients.* (G1, P1, NUR) 

*Until today, after six months of the pandemic, I have not carried out any serology tests to find out if I have already had contact with the virus, and several colleagues have already tested positive near us.* (G3, P4, NT) 

*I didn’t go to work contaminated, but two of my period got contaminated. No examination was carried out.* (G3, P5, NT) 

#### Concern for patients, colleagues and managers

According to research participants, the need for complex and permanent care for patients with COVID-19, concerns about the overload of co-workers and pressure from managers and institutions were factors that led nursing professionals to work even when they are sick. According to the interviewees, the pandemic increased the number of professionals on leave, which, together with the chronic lack of substitutes, intensified presenteeism.

*We still work sick sometimes, because we don’t want to miss the scale and overload our friends, since we work with a small number of employees.* (G2, P4, NT) 

*Yes, I went to work with the virus, all so as not to burden my colleagues, as the team was already reduced.* (G4, P1, NT) 

*We had no employees, we were all spoiled, there was no way to say “I won’t” and let the team down.* (G6, P2, NT) 

*I think that weighs a lot, thinking that if we don’t go, we’re going to harm those who are there, and the fear of being frowned upon by the boss.* (G1, P1, NUR) 

*Yes, I went to work with an inflamed sciatic nerve (...) and there was no one at the time to replace me.* (G2, P3, NT) 

*It has two sides: I need to work, and a feeling that the team, my boss, and the people need my work.* (G6, P5, NUR) 

#### Fear of losing job or financial benefits

The interviewees criticized the difficulties encountered in obtaining medical certificates or consolidating absences at work, as well as the fear of losing “benefits”, such as food vouchers and access to extra shifts. The participants stated that the presentation of a medical certificate was commonly seen by the employing institutions as an “excuse” for workers to be absent. Consequently, the fear of losing their job in case of absenteeism became latent, leading them to a presentist work culture.

*On my first day at work, I was told that a good employee is the one who doesn’t have a certificate. Each time you take a certificate, your food allowance is deducted. Not to mention that many people have already been fired for taking a certificate.* (G1, P1, NT) 

*I already went to work with a headache for a week, because I didn’t want to lose my extra shifts with a certificate.* (G4, P2, NUR) 

*Several times I’ve worked sick. When a certificate was taken, we were threatened with losing our shift.* (G2, P4, NT) 

*When this happens (presenteeism) we have the impression that it is the longest shift of our lives, but it still ends up being done to avoid possible discounts on payslips or conflicts on the part of management who think it is an excuse.* (G5, P4, NT) 

*I’ve already gone to work sick several times so as not to take risks when I have a cut, to avoid being fired.* (G1, P5, NT) 

*I worked anyway for fear of losing work! Because the company, when it doesn’t need you, fires you and simply replaces you!* (G2, P1, NT) 

### Old problems, permanent struggle

#### In addition to the honors

Despite the role of nursing in combating the COVID-19 pandemic, the professionals interviewed reported that media, government and society tributes and thanks did not contribute to improving their harsh reality. The various forms of precarious work remained latent, including the chronic deficit of human resources, low wages, the risk of dismissal, the loss of labor rights, work-related violence and the reduction of government investments in health and education in Brazil.

*Professional achievement comes first from the recognition of work. We are experiencing the pandemic that has greatly highlighted the “heroism” of health professionals, however, we are now living in a moment of layoffs and the end of contracts. Not to mention the low wages, work overload, embezzlement of scales, etc.* (G6, P4, NUR) 

*There is a lot of talk about health professionals, but recognition beyond words does not exist.* (G4, P2, NUR) 

*It doesn’t matter pots, it doesn’t matter the message of “we are with you”, what really matters is what will be done to cure these sick professionals, who will pay for our rights that were taken away. (...) Honestly, I don’t see that this voice will be heard by a population that attacks a health professional on public transport out of fear that he, a worker, will pass on the virus while this aggressor is going to a clandestine party. Where rulers approve freezing funding in health and education. If they did this before the pandemic, why would they change it now?* (G4, P3, NT) 

#### Nursing calls for a new reality

The professionals interviewed expressed the need for free psychological support, safe working environments, adequate material and organizational inputs for assistance, permanent education, channels for listening to workers, fair remuneration, adequate proportion between patients assisted and professionals hired, resting environments in the work and career plans. The needs for valuing and humanizing nursing professionals, both inside and outside the workspace, were cited. Finally, the workers highlighted the importance of collective struggle in the achievement of labor rights, such as greater investments in health, institution of the minimum wage for nursing and regulation of the 30-hour week of work.

*Proper delivery of PPE [Personal Protective Equipment] and psychological support would be of great help!* (G1, P5, NT) 

*(...) It should count and value our specializations and postgraduate courses.* (G6, P5, NUR) 

*Adequate remuneration, fair workload, an adequate team with the right amount of professionals, a rest room for 12-hour shifts...* (G5, P3, NUR) 

*I think that each institution should have humanization with the employees, because humanization with the patient is preached a lot, but we offer what we receive.* (G6, P2, NT) 

*Adequate and functioning work equipment, training or continuous and permanent training, the importance of co-participatory management ... Another thing we are looking for is the approval of a national wage floor for the category and a 30-hour workweek.* (G4, P3, NT) 

*And finally, among all this, PL [Bill] 2564, which gives us a glimmer of hope, but which we know will not be easy. I think that now is the time, hardly in another sad moment like this will nursing be on the agenda in this way.* (G4, P1, NT) 

## Discussion

The present study identified experiences of worsening presenteeism among nursing professionals in the context of the COVID-19 pandemic. Respondents reported institutional pressure to return to work even when ill, mental suffering and feelings of devaluation and dehumanization at work despite their efforts in a critical context. The lack of testing for the disease, concern for colleagues, patients and managers, as well as the fear of losing their job or financial benefits, led professionals to presenteeism. Given this scenario, nursing pointed out the urgent need to be recognized beyond honors, through the guarantee of decent wages and safe working conditions.

Presenteeism can intensify in jobs with high pressure, work overload and whose work culture stigmatizes sick leave, a situation that already existed and has worsened in the context of the COVID-19 pandemic ^(^ 21 ^)^. Individuals may continue to work or return to work earlier when they believe that their managers or co-workers do not consider them “sick enough” to abstain ^(^ 22 ^)^. This institutional pressure for presenteeism may be even greater in the case of workers who received a negative diagnosis of COVID-19 or developed mild symptoms of the disease ^(^ 22 ^)^. However, it is known that COVID-19 can cause persistent symptoms, such as chronic fatigue, weakness and cognitive changes ^(^ 22 ^)^. 

The workers’ mental suffering was also linked to presenteeism. A scope review on situations of psychological distress among nursing professionals in the COVID-19 pandemic indicated its relationship with work overload, lack of PPE and fear of getting sick, infecting others or acting in direct patient care diagnosed or with suspicion of the disease ^(^ 23 ^)^. The most common signs and symptoms of psychic suffering were anxiety, sadness, insomnia, stress and fear ^(^ 23 ^)^. 

Among the factors that led nursing professionals to presenteeism, the concern with colleagues, patients and managers stood out in this study. International studies developed in the pandemic context, in countries such as Portugal, Switzerland and the United States, have also pointed to this relationship ^(^ 21 ^-^ 24 ^)^. A culture of self-sacrifice at work and a strong sense of loyalty and responsibility for the well-being of others, linked to increased pressure on care and a shortage of employees, can exacerbate presenteeism ^(^ 21 ^,^ 24 ^-^ 25 ^)^. 

Fear of losing a job or financial benefits was also mentioned as an influencing factor for presenteeism among nursing workers. A study with health professionals in Israel identified that most respondents reported working sick due to a punitive institutional culture ^(^ 26 ^)^. It is noteworthy that such management strategies were strengthened in a Brazilian scenario of deleterious structural reforms, among them labor and social security reforms, which are direct examples of the erosion of labor rights in Brazil ^(^ 27 ^)^. 

The lack of tests for detecting COVID-19 was also identified as a trigger for presenteeism among nursing professionals. According to the World Health Organization (WHO), extensive testing is defined as one of the pillars for disease control ^(^ 28 ^)^. It is known that the late recognition of COVID-19 cases among health professionals can lead to presenteeism and violations in infection control, contributing to nosocomial outbreaks of COVID-19 ^(^ 29 ^)^. 

The lack of systematic testing of COVID-19 cases in Brazil highlighted one of the several negligences in the management of the pandemic by the Federal Government ^(^ 9 ^-^ 11 ^)^. The absence of national protocols and guidelines, lack of inputs, shortage of asymptomatic testing, disparity in the distribution of tests and low quality of reporting systems stood out in the country ^(^ 10 ^)^. In addition, the country was affected by the disorderly and late acquisition of vaccines available for disease prevention, in a scenario of discourses of denial of the severity of COVID-19 ^(^ 9 ^-^ 11 ^)^. Political leaders, whose decision-making power influenced the inequity of the population’s access to protective resources against the disease, strengthened the spread of unsafe information without scientific proof ^(^ 11 ^)^. 

Given the scenario presented, nursing professionals highlighted a strong sense of devaluation of their work. Feelings of non-recognition of their role in coping with the pandemic and dehumanization at work were expressed by feeling like “robots” or not being treated like human beings, which is similar to debates brought up in other studies ^(^ 8 ^-^ 24 ^)^. 

During the COVID-19 pandemic, a false romanticization of nursing work was observed in Brazil, seen as priesthood or heroism, which was not reflected in real changes in working conditions. As a more brutal example, nursing professionals were subject to violence at work, whether by defending scientifically legitimized protection and assistance measures, or by being seen as a source of contamination ^(^ 30 ^-^ 31 ^)^. 

The severity of the COVID-19 pandemic and the number of deaths among nursing professionals in Brazil were not enough for minimum wage rights to be guaranteed. For more than a decade, Brazilian nursing has been fighting for the institution of a national wage floor for the category ^(^ 32 ^)^. Although approved as a law in 2022, its application was not implemented and continues to be hit by neoliberal barriers on the part of companies and medical-hospital organizations, based on arguments of lack of funding sources and the threat of reducing beds and mass layoffs ^(^ 32 ^)^. 

The performance of nursing in a highly critical context such as the COVID-19 pandemic, intensely marked by presenteeism, highlights the precariousness of nursing work in Brazil, governed by a neoliberal perspective that seeks to use labor until its wear and tear final, refraining from caring for those who care.

In this sense, it is essential that employing institutions guarantee better health and safety conditions at work, especially in pandemic contexts, with free provision of diagnostic tests to workers and clear messages that encourage them to stay at home when sick, without threats or harm to their jobs ^(^ 3 ^)^. Above all, it is essential to ensure the social participation of workers, who in the current Brazilian scenario demand the establishment of a decent wage floor ^(^ 32 ^)^ without which decent work will not be achieved. 

As limitations of this study, it should be noted that comparative analyzes of presenteeism were not developed between nurses and nursing technicians and between public and private hospital contexts, which could generate more robust reflections on the studied phenomenon. Furthermore, data collection was restricted to the experiences of nursing professionals from only two of the five Brazilian regions.

However, it is considered that the results of this research can contribute to the advancement of knowledge in the area of health and nursing, as they reveal the lived experiences, the factors related to presenteeism in a pandemic context and the urgent demands of the class, to that this labor category gains appreciation and rights that have been claimed for some time.

## Conclusion

Nursing professionals expressed a worsening of presenteeism during the COVID-19 pandemic, marked by institutional pressure to return to work, mental suffering, devaluation and dehumanization at work. Lack of access to disease detection tests, concern for patients, co-workers and managers, as well as fear of losing employment or financial benefits resulted in presenteeism. Workers also pointed out the need for an appreciation of the profession that is not limited to mere speeches or tributes.

Thus, it was concluded that presenteeism during the pandemic context manifested itself within a neoliberal social, economic and political conjuncture in which the health and life of workers were less relevant than the accumulation of capital. That said, nursing workers demand adequate human and material resources, a reduction in workload, the institution of a minimum wage and recognition and humanization at work.

## References

[ref-1] Zuzelo PR (2017). Going to Work While Sick: The Phenomenon of Sickness Presenteeism. Holist Nurs Pract [Internet].

[ref-2] Min A, Kang M, Park H (2022). Global prevalence of presenteeism in the nursing workforce: A meta-analysis of 28 studies from 14 countries. J Nurs Manag [Internet].

[ref-3] Daniels S, Wei H, Han Y, Catt H, Denning DW, Hall I (2021). Risk factors associated with respiratory infectious disease-related presenteeism: a rapid review. BMC Public Health [Internet].

[ref-4] The Lancet (2020). COVID-19: protecting health-care workers. Lancet [Internet].

[ref-5] Semple S, Cherrie JW (2020). Covid-19: Protecting Worker Health. Ann Work Expos Health [Internet].

[ref-6] Conselho Federal de Enfermagem (BR) (2023). Observatório da Enfermagem [Homepage].

[ref-7] Freire NP, Castro DA, Fagundes MCM, Ximenes FRG, Cunha ICKO, Silva MCN (2021). News on Brazilian Nursing in the COVID-19 pandemic. Acta Paul Enferm [Internet].

[ref-8] The Lancet (2023). The future of nursing: lessons from a pandemic. Lancet [Internet].

[ref-9] Sodré F (2020). COVID-19 epidemic: critical issues for public health management in Brazil. Trab Educ Saúde [Internet].

[ref-10] Lopes MLDS, Lima KC (2021). The COVID-19 pandemic and shortcomings in management approach on a population level. Rev Bras Geriatr Gerontol [Internet].

[ref-11] Maciel E, Fernandez M, Calife K, Garrett D, Domingues C, Kerr L (2022). The SARS-CoV-2 vaccination campaign in Brazil and the invisibility of science evidences. Cien Saude Colet [Internet].

[ref-12] Fonseca EM, Nattrass N, Lazaro LLB, Bastos FI (2021). Political discourse, denialism and leadership failure in Brazil’s response to COVID-19. Glob Public Health [Internet].

[ref-13] Pereira F, Querido AI, Bieri M, Verloo H, Laranjeira CA (2021). Presenteeism Among Nurses in Switzerland and Portugal and Its Impact on Patient Safety and Quality of Care: Protocol for a Qualitative Study. JMIR Res Protoc [Internet].

[ref-14] Araújo WRM, Siqueira AMO (2021). Dialectical historical materialism and the historicity of society in Marx (1818-1883). https://rsdjournal.org/index.php/rsd/article/view/12012.

[ref-15] Antunes R (2022). Capitalismo Pandêmico.

[ref-16] Vinuto J (2014). Snowball sampling in qualitative research: an open debate. Temat [Internet].

[ref-17] Moura CO, Silva IR, Silva TP, Santos KA, Crespo M CA, Silva MM (2022). Methodological path to reach the degree of saturation in qualitative research: grounded theory. Rev Bras Enferm [Internet].

[ref-18] Oliveira JC, Penido CMF, Franco ACR, Santos TLAD, Silva BAW (2022). The specificities of the online focal group: an integrative review. Cien Saude Colet [Internet].

[ref-19] Abreu NR, Baldanza RF, Gondim SMG (2009). Focal groups on-line: from the conceptual reflections to the virtual environment application. J Inf Syst Technol Manag [Internet].

[ref-20] Minayo MCS (2014). O desafio do conhecimento: pesquisa qualitativa em saúde.

[ref-21] Kinman G, Grant C (2021). Presenteeism during the COVID-19 pandemic: risks and solutions. Occup Med (Lond) [Internet].

[ref-22] Wise J (2020). Long covid: doctors call for research and surveil-lance to capture disease. Br Med J [Internet].

[ref-23] Miranda FBG, Yamamura M, Pereira SS, Pereira CS, Protti-Zanatta ST, Costa MK (2021). Psychological distress among nursing professionals during the COVID-19 pandemic: Scoping Review. Esc Anna Nery [Internet].

[ref-24] Laranjeira C, Pereira F, Querido A, Bieri M, Verloo H (2022). Contributing Factors of Presenteeism among Portuguese and Swiss Nurses: A Qualitative Study Using Focus Groups. Int J Environ Res Public Health [Internet].

[ref-25] Santos DGSM, Conceição AAM, Ferreira MMF (2022). Presenteeism in healthcare workers on a pandemic context by COVID-19 disease: A scoping review. Rev Enf Ref [Internet].

[ref-26] Gur-Arie R, Katz MA, Hirsch A, Greenberg D, Malosh R, Newes-Adeyi G (2021). “You Have to Die Not to Come to Work”: A Mixed Methods Study of Attitudes and Behaviors regarding Presenteeism, Absenteeism and Influenza Vaccination among Healthcare Personnel with Respiratory Illness in Israel, 2016-2019. Vaccine [Internet].

[ref-27] Antunes R (2020). Uberização, Trabalho Digital e Indústria 4.0.

[ref-28] World Health Organization (2022). WHO policy brief: COVID-19 testing [Internet]. https://www.who.int/publications/i/item/WHO-2019-nCoV-Policy_Brief-Testing-2022.1.

[ref-29] Eisen D (2020). Employee presenteeism and occupational acquisition of COVID-19. Med J Aust [Internet].

[ref-30] Ribeiro BMSS, Robazzi MLCC, Dalri RCMB (2021). Violence caused to health professionals during the COVID-19 pandemic. Rev Saúde Pública Paraná [Internet].

[ref-31] Alves JS, Gonçalves AMS, Bittencourt MN, Alves VM, Mendes DT, Nóbrega MPSS (2022). Psychopathological symptoms and work status of Southeastern Brazilian nursing in the context of COVID-19. Rev. Latino-Am. Enfermagem [Internet].

[ref-32] Peduzzi M (2022). The various meanings of the rejection to the nursing basic remuneration floor. Rev Paul Enferm [Internet].

